# Cancer care in the COVID-19 era and psychosocial impacts on oncology nursing in Brazil

**DOI:** 10.3332/ecancer.2021.1331

**Published:** 2021-12-09

**Authors:** Tamara Otsuru Augustinho Teixeira, Leticia Gomes Carvalho, Guilherme Gasparini Camargo, Edvane Birelo Lopes De Domenico

**Affiliations:** 1 Escola Paulista de Enfermagem, Universidade Federal de São Paulo, 754, Napoleão de Barros Street, Vila Clementino, São Paulo, SP 04023-062, Brazil; 2 Universidade Federal de São Paulo, 754, Napoleão de Barros Street, Vila Clementino, São Paulo, SP 04023-062, Brazil; 3 Universidade Federal de São Carlos, Washington Luiz, s/n – São Carlos, SP 13565-905, Brazil; 4 Department of Clinical and Surgical Nursing, Escola Paulista de Enfermagem, Universidade Federal de São Paulo, 754, Napoleão de Barros Street, Vila Clementino, São Paulo, SP 04023-062, Brazil; a https://orcid.org/0000-0002-3588-653X; b https://orcid.org/0000-0003-1719-6014; c https://orcid.org/0000-0003-4310-892X; d https://orcid.org/0000-0001-7455-1727

**Keywords:** nursing, medical oncology, coronavirus infections, psychosocial impact, pandemics

## Abstract

**Introduction:**

Globally, Brazil has the third highest number of confirmed COVID-19 cases and the second highest number of deaths related to COVID-19 at the time of writing. Maintaining cancer care has been a challenge for patient safety and for the physical and mental health of oncology nurses.

**Objectives:**

To describe which effects of the COVID-19 pandemic on cancer care could already be evaluated and to identify the psychosocial impact on cancer nurses in Brazil.

**Method:**

Reflective, analytical, qualitative study.

**Results:**

Although the Brazilian cancer care policy has reached important achievements in overcoming access barriers to cancer diagnosis and treatment over the past decade, the COVID-19 pandemic has caused losses of timely access to health services for patients with cancer, which has compromised screening, early diagnosis and treatment, and patient follow-up. Oncology nurses have actively participated in the management and assistance strategies during the pandemic. This path has generated an increase in oncology nurses’ workload, leading to physical and mental stress and anxiety related to the fear of contagion for themselves and their family.

**Conclusion:**

The pandemic has affected the care of cancer patients, with the potential to suffer greater losses because of reductions in screening, early diagnosis and treatment, and patient follow-up. In addition to the worsening of the cancer setting, the COVID-19 pandemic has overwhelmed Brazilian oncology nurses, physically and mentally.

## Introduction

The COVID-19 pandemic has generated uncertainties and concerns worldwide in various social sectors, due to destabilisation of the existing order and unpredictability about the future. In the health sector, the COVID-19 pandemic has mobilised the system to face the risks of contagion from severe acute respiratory syndrome coronavirus 2 (SARS-CoV-2) and to search for better evidence of treatment for patients. In addition, the pandemic has changed assistance protocols at the primary, secondary and tertiary levels of all acute and chronic diseases [1, 2].

As one of the four non-communicable chronic diseases with the greatest global impact, cancer has a prominent position for measures to protect patients’ rights to care in a pandemic condition [3, 4]. Many actions have been taken to mitigate damage to patients with cancer and most have been under nurse administrative and assistance responsibilities, mainly nurses who are specialised in critical care (intensive or emergency care) or chronic diseases, such as oncology nurses, among other specialties. In consequence, nurses working on the front line, in a few months of the pandemic, already had physical and mental changes that were typical of distress situations [5–7].

Brazil has 20,350,142 confirmed cases of COVID-19 – the third highest number in the world, and also has the second highest number of deaths with 568,788 deaths due to COVID-19, as of 15 August 2021 [8]. Patients in Brazil can access health care through private services and/or the Brazilian Unified Health System (SUS), the Brazilian public health system. SUS was created in 1988, inspired by the UK National Health Service, but unfortunately, there are significant differences regarding timely access, resources and patient outcomes between SUS and private services [9]. Currently, about 7/10 Brazilians, or more than 150 million people, depend exclusively on SUS for health care, showing the importance of this service for the Brazilian population [10].

The Brazilian national policy to prevent and control cancer, launched in 2013, included principles and guidelines related to promotion, prevention, education, communication, incorporation of technology, and responsibility in cancer control actions, aiming at care comprehensiveness in SUS [11]. This led to improvements in the quality of the cancer care offered by SUS, such as a requirement to initiate treatment for patients with neoplasia within a maximum period of 60 days from diagnosis. There was also wider distribution of radiation therapy technology, expansion of centres with several therapeutic modalities and municipality-based decentralisation [12].

National studies have shown the difficulties of controlling the dissemination of SARS-CoV-2 in Brazil, especially in communities with high population density where people live without basic sanitation and face difficulties in receiving medical care. These communities have shown the highest transmission rates because of marked multiple generational living conditions and large families. Additionally, this pandemic has had a strong negative impact on the country’s economy, and predictable consequences related to greater educational delay for children and adolescents. In the health area, there have been losses in diagnosis and treatment of other diseases because of the priority given to care related to COVID-19 [13, 14].

COVID-19 has imposed new rules and, despite efforts to continue cancer care, the results are still unknown. The literature has estimated a negative impact on cancer outcomes in Brazil in the near future, due to alterations in the screening programme alone [15].

The scenario of cancer care and the COVID-19 pandemic also has psychosocial repercussions for Brazilian oncology nurses working in oncology. Thus, we aimed to describe which effects of the COVID-19 pandemic on cancer care could already be evaluated and identify the psychosocial impact on cancer nurses in Brazil.

## Method

This study is a narrative literature review, and these steps were followed: elaboration of guiding questions, sample selection, analysis of extracted data and synthesis of knowledge. The guiding questions were: What effects could already be evaluated in cancer care as a result of the COVID-19 pandemic in Brazil? How have Brazilian oncology nurses reacted psychosocially during the pandemic?

We searched LILACS, PubMed, MEDLINE, SciELO, Google (for grey literature) and websites of Brazilian governmental and non-governmental health and cancer care societies and entities.

The search terms were the Medical Subject Headings (MeSH) Nursing, Cancer, Neoplasms, Epidemiology, Medical Oncology, Coronavirus Infections, Psychosocial Impact, Pandemics and Brazil, used in combination, for literature published between 1 March 2020, and 30 April 2021. Papers published in English and Portuguese were reviewed and the final reference list was generated based on the relevance to the scope of this review.

Data was analysed along with the guiding questions and generated the themes – cancer care and the COVID-19 pandemic: a sum of challenges and psychosocial impact on Brazilian cancer nursing: burdens and gains.

### Cancer care and the COVID-19 pandemic: a sum of challenges

There were widespread efforts by Brazilian health regulatory agencies to ensure that no chronic disease treatments were interrupted. In addition to primary health care restructuring to maintain access to general health care, cancer treatment centres in tertiary care were rationalised with new care protocols, educational programs and flows oriented towards patient safety, based on international and Brazilian recommendations to mitigate the propagation of SARS-CoV-2. Factors such as disease severity, potential benefits of treatment, immunosuppression potential of medication regimens, patient age and previous conditions began to be considered because of the risks posed by COVID-19 [16, 17]. A decrease of 70% for oncology surgery and 50%–90% of biopsies for pathology were noted. Based on this data, an estimated 50,000 Brazilians have not received a cancer diagnosis during the two first months of COVID-19 pandemic alone [18].

The *Oncoguia* Institute, one of the leading non-governmental organizations fighting cancer in Brazil, conducted an online survey from 15 March to 10 May 2020. With 566 respondents, including patients (89%) and families (9%) from all Brazilian regions, 43% indicated that the COVID-19 pandemic had affected their cancer treatment (60% SUS patients, 33% private and 53% users using both services). When asked about what aspects of their lives had been impacted, 32% said financial and 52% emotional [19].

The Brazilian Society of Clinical Oncology also conducted a survey. Of 120 oncologists from both public and private services, 74% said one or more patients had interrupted or postponed their treatment for more than 1 month during the pandemic. The most frequently postponed services were surgery (67%) and follow-up exams (22.5%); only 0.83% believed their patients had not faced any problems [20].

In addition, aiming at unravelling the scenario in secondary care, a study that compared the number of cancer diagnoses registered by the SUS before and during the pandemic found a relevant decrease in all Brazilian regions, ranging from −24.3% in the North to −42.7% in the Northeast in 2020. The Brazilian general deficit average reached 35.5%, which means that 15,000 cases of cancer per month were not diagnosed [21].

Thus, despite international oncological societies and the Brazilian Ministry of Health advising patients with cancer to continue treatment, factors such as insufficient flows and problematic structural, material and human resources have rendered adequate cancer care unviable at all levels [22].

From the perspective of patients with cancer affected by COVID-19, a study by the Brazilian National Cancer Institute (INCA) from 30 April to 26 May 2020 with patients treated by SUS concluded that the rates of COVID-19 related complications and deaths were significantly high [23].

Therefore, huge efforts will be needed to mitigate the quantitative and qualitative damage caused by the COVID-19 pandemic in Brazilian cancer care at all levels of care, from primary attention to rehabilitation.

### Psychosocial impact on Brazilian cancer nursing: burdens and gains

In a study of the impact of COVID-19 on patients with cancer in low- and middle-income countries, the most common concerns of Brazilian oncology nursing were insufficient personal protective equipment (PPE), interruption of patients’ treatment and suffering caused by the uncertainty, the fear of taking the coronavirus to their loved ones and stressful workload [24]. Despite the resources and healthcare system differences that exist among Jordan, India and Ghana, Brazilian oncology nursing seems to have the same concerns as oncology nurses from the countries represented in this study.

Unfortunately, patients with cancer and COVID-19 infection have had a deterioration of their clinical condition and sometimes deaths. Co-workers have also been infected and despite the absence of risk factors, some of them developed severe disease and recovered with sequelae. Infected colleagues that lived with their parents, ended up infecting them as well; some of these professionals had to manage their parent’s admission to an ICU and there were cases when the parent passed away.

A qualitative study performed at a hospital specialised in cancer treatment in Rio de Janeiro describe the challenges of nursing management in oncology intensive care during the COVID-19 pandemic and measures adopted to manage the disease [25]. The authors highlighted the need for frequent changes in nursing shifts due to increased absenteeism, COVID-19 disease or other diagnoses related to an overload of physical and mental work caused by caring for patients with COVID-19 [25].

Another possible stress-generating factor for oncology specialist nurses was the change in work organization, imposing new learnings, with little time for dedication to study or training. In this new order, the most emblematic example is perhaps switching from in person and home care nursing appointments to virtual ones, as highlighted in a study that reported changes in palliative care for patients with advanced cancer and COVID-19 in the Hospital do Câncer IV do Instituto Nacional de Câncer José Alencar Gomes da Silva (INCA), in Rio de Janeiro [26].

For Brazilian nurses, consultation by phone or on virtual platforms was not standardised or authorised by the Federal Council of Nursing until the COVID-19 pandemic [27]. Although it is believed that, if well monitored, tele-consultation can strengthen cancer care during COVID-19 pandemic by ensuring social isolation, reducing mobility costs and providing early detection and intervention for disease and treatment complications [28], a gap in the scientific evidence remains to determine if this alternative is acceptable and helpful for patients in regard to therapeutic adherence, resilience, efficient management of symptoms and toxicities, quality of life and well-being, in addition to strengthening the patient–family–nurse bond. The complexity of this approach was demonstrated in a pre-pandemic study of patients with lung cancer receiving an intervention to improve coping skills in the US [29].

Concerned about the impact of this pandemic on nurses, the Brazilian Federal Council of Nursing launched the ‘Solidary Nursing Program’, providing nurses with safety guidelines resources and a 24 hours/7-day online chat for mental health support, from 26 March to 20 September 2020. During this period, this initiative provided more than 5,000 appointments. As the shortage of PPE was solved, the nurses’ concern about their families’ support and contagion were the main cause of their intense distress. A fear of death and suffering from physical and mental exhaustion had become common due to patients, co-workers, families, and friends’ deaths [30].

A similar mental impact of COVID-19 can be seen in the oncology workforce worldwide. Among 1,520 oncology professionals from 101 countries who participated in a global online survey #I conducted by the European Society for Medical Oncology, from 16 April to 3 May 2020, 25% of the participants were at risk of distress, 38% reported feeling burnout and 66% not being able to perform their job compared with the pre-COVID-19 period. In survey #II, conducted from 16 July to 5 August 2020, among 272 professionals who completed both surveys, well-being scores ≥4 and greater and burnout rates were significantly higher compared with survey I (22% versus 31%, *p* = 0.01; and 35% versus 49%, *p* = 0.001, respectively), suggesting well-being and burnout have worsened over a 3-month period during the COVID-19 pandemic [31].

Strategies for controlling the COVID-19 pandemic must address well-being and improve resilience of oncology professionals [31] as psychological assistance, mainly to oncology nurses as they are part of the most susceptible groups for psychological suffering [32]. Oncology professionals must be able to recognise the signs of psychological distress in self and among colleagues, such as depression, anxiety and burnout to favour early intervention. A safety, supportive psychological environment, with open, transparent and private communication at leadership level should be available [33].

Although this pandemic has imposed social distancing, technology has had the power to make us feel close to each other. National societies specialised in oncology, supported by several health communication companies, pharmaceuticals and medical devices industries, have been responsible for organising and maintaining, live weekly interviews with expert professionals linked to the most important healthcare, teaching and educational institutions, from all Brazilian regions in live sessions, with the possibility of attendee’s question and answer sessions. The live webinars and on-demand resources had democratised continuing education that is so essential and at the same time so challenging in a country with the continental size and disparities as Brazil. These new formats are providing us with the opportunity to connect with oncology nursing worldwide, exchange knowledge and experiences and become up to date about the state of the art of cancer care, a good way to keep our minds busy and productive [34].

And so, the experience report of an oncology nurse from a cancer hospital in Rio de Janeiro, seems to be the experience that many Brazilian oncology nurses are facing during COVID-19 times: ‘relearn with science, technologies in health care, health education and disease in order to foresee and act, extracting from this experience a singular care construction that must be accompanied, studied and reframed’ [35].

The limitations of this study derive from the type of study, reflective-analytical, based on mixed, scientific and grey literature.

## Conclusion

Cancer is a public health problem in low- and middle-income countries that is aggravated by social inequities and resource scarcity. The COVID-19 pandemic has affected the care of patients with cancer, with the potential to suffer greater losses because of reductions in screening, early diagnosis and treatment, and patient follow-up. Additionally, patients with cancer and SARS-CoV-2 have been documented to have a higher mortality rate than those with cancer alone.

Oncology nurses are managing the sum of uncertainties generated by this pandemic, the risk of being infected by COVID-19 and the perception of the epidemiological, clinical and emotional consequences for patients with cancer and their families. They are also experiencing physical and psychological overload caused by exhaustive work on the front line.

This reality should mobilise health managers to build plans capable of mitigating the harm caused to patients in the present and favour a psychosocial support network capable of supporting oncology nurses. In this way, despite all the difficulties, oncology nurses can transcend the pandemic and emerge more competent, self-effective and self-realised from this experience.

## Conflicts of interest

The authors declare that they have no conflicts of interest.

## Funding

No funding was obtained for this study.

## Authors’ contributions

TOAT and EBLDD were responsible for the design of the present study. LGC and GGC were involved in data collection. All authors were involved in data analysis and/or interpretation. All authors read and approved the manuscript. EBLDD was the project advisor.
